# Cardioprotective effects of adenosine within the border and remote areas of myocardial infarction

**DOI:** 10.1186/2191-219X-3-65

**Published:** 2013-09-12

**Authors:** Mélanie Bousquenaud, Fatiha Maskali, Sylvain Poussier, Jennifer Zangrando, Pierre-Yves Marie, Henri Boutley, Renaud Fay, Gilles Karcher, Daniel R Wagner, Yvan Devaux

**Affiliations:** 1 Laboratory of Cardiovascular Research, Centre de Recherche Public de la Santé, 84 Val Fleuri, Luxembourg L1526, Luxembourg; 2 Nancyclotep Experimental Imaging Platform, 54 511, Nancy, France; 3 INSERM, U1116, 54 000, Nancy, France; 4 INSERM, Centre d'Investigation Clinique CIC-P 9501, Nancy 54000, France; 5 Division of Cardiology, Centre Hospitalier, Luxembourg L-1210, Luxembourg

**Keywords:** Adenosine, Myocardial infarction, Contractility, Left ventricular remodelling, Gated cardiac positron emission tomography

## Abstract

**Background:**

Adenosine may have beneficial effects on left ventricular function after myocardial infarction (MI), but the magnitude of this effect on remote and MI areas is controversial. We assessed the long-term effects of adenosine after MI using electrocardiogram-triggered ^18^ F-fluorodeoxyglucose positron emission tomography.

**Methods:**

Wistar rats were subjected to coronary ligation and randomized into three groups treated daily for 2 months by NaCl (control; *n* = 7), 2-chloroadenosine (CADO; *n* = 8) or CADO with 8-sulfophenyltheophilline, an antagonist of adenosine receptors (8-SPT; *n* = 8).

**Results:**

After 2 months, control rats exhibited left ventricular remodelling, with increased end-diastolic volume and decreased ejection fraction. Left ventricular remodelling was not significantly inhibited by CADO. Segmental contractility, as assessed by the change in myocardial thickening after 2 months, was improved in CADO rats compared to control rats (+1.6% ± 0.8% vs. −2.3% ± 0.8%, *p* < 0.001). This improvement was significant in border (+5.6% ± 0.8% vs. +1.5% ± 0.8%, *p* < 0.001) and remote (−4.0% ± 1.0% vs. −10.4% ± 1.3%, *p* < 0.001) segments, but absent in MI segments. Histological analyses revealed that CADO reduced fibrosis, cardiomyocyte hypertrophy and apoptosis. Protective effects of CADO were blunted by 8-SPT.

**Conclusion:**

Long-term administration of adenosine protects the left ventricle from contractile dysfunction following MI.

## Background

Despite reperfusion therapies, left ventricular (LV) remodelling occurs in many patients after acute myocardial infarction (MI), leading to a risk of subsequent heart failure [1]. This has stimulated efforts to develop new pharmacological strategies to prevent or reverse LV remodelling. LV remodelling is defined as a complex sequence of changes in left ventricle geometry and function [2,3] involving progressive LV dilatation, cardiomyocyte hypertrophy and fibrosis, ultimately leading to the loss of contractile function and LV dysfunction. The long-term outcome of acute MI patients largely depends on the amount of the initial injury which conditions subsequent LV remodelling. Thus, therapeutic strategies aiming at reducing infarct size and improving LV remodelling are of major interest.

Adenosine is a ubiquitous endogenous nucleoside that modulates physiological functions in various organs and plays important roles within the cardiovascular system. It can signal through four sub-types of G protein-coupled receptors, all of which are expressed in the heart [4]. Cardioprotective properties of adenosine have been characterized, particularly during ischemic pre-conditioning. However, the long-term effect of adenosine after MI has received less attention.

Disappointing results were documented when adenosine was administrated during the acute phase of reperfused MI in humans [5-7]. However, further experimental data suggest that beneficial effects could be achieved through a long-term administration. In particular, long-term administration of A2B adenosine receptor agonist in a rat infarct model improved global LV function assessed by echocardiography [8]. A recent study showed that infarct size could be reduced by increasing adenosine levels in transgenic swine through over-expression of ectonucleoside triphosphate diphosphohydrolase-1 (CD39) [9].

While adenosine appears to have certain beneficial effects after MI, the mechanisms and sites of these effects, as well as the consequences on global LV remodelling, are poorly documented. Therefore, the aim of the current study was to investigate the effects of long-term administration of adenosine on LV function and remodelling after MI by serial non-invasive imaging with electrocardiogram (ECG)-triggered ^18^ F-fluorodeoxyglucose positron emission tomography (FDG-PET). FDG-PET has been reported as an imaging technique for assessing the severity and the location of MI and is also capable of quantifying global and segmental LV function [10,11]. Thus, FDG-PET allows for a separate analysis of the contractile function of MI, remote and border segments.

## Methods

This study was performed in accordance with the regulations of the Animal Welfare Act of the National Institutes of Health Guide for the Care and Use of Laboratory Animals (NIH Publication No. 85-23, revised 1996), and protocols were approved by the Regional Veterinary Department (‘Direction Départementale de la Protection des Populations’), agreements RAR1A03516811825 and 54–100.

### Animal model and experimental design

Twenty-three adult male Wistar rats (282- to 335-g body weight at the beginning of the study; Charles Rivers Laboratories, Wilmington, MA, USA) were enrolled. All animals were housed on a 12-h light-dark cycle in a room with temperature and humidity control, and with *ad libitum* access to tap water and standard rodent food. Permanent occlusion of the left anterior descending (LAD) coronary artery was performed in 23 rats as previously described [10,12]. The choice of this model rather than a model of ischemia/reperfusion was based on preliminary experiments suggesting that adenosine may be more protective in large infarcts than small infarcts (not shown). Rats were anaesthetized by inhalation of an isoflurane/oxygen mixture (2% to 3%:1.5% *v*/*v*) and were intubated and ventilated with a rodent ventilator (Harvard Apparatus, Holliston, MA, USA). The heart was exposed through a left lateral thoracotomy of the fifth intercostal space. After pericardial incision, the proximal part of the LAD (2 to 3 mm from the top of the left atrium) was ligated with a 7-0 Prolene suture (Ethicon, Somerville, NJ, USA). Perioperative lidocaine (10 mg/kg, Aguettant, Lyon, France) was used to provide a local anaesthesia and to reduce the incidence of ventricular tachycardia and fibrillation. Finally, the chest was closed in layers with a 2-0 Vicryl suture (Ethicon, Somerville, NJ, USA), the lungs were reinflated using positive-end expiratory pressure, and the endotracheal tube was removed once spontaneous breathing had resumed. Amoxicillin was injected intramuscularly (60 mg/kg, GlaxoSmithKline, Marly-le-Roi, France) to avoid bacterial infections. The mortality of the procedure was <20%.

Two days after surgery, rats were assessed by ECG-triggered ^18^ F-FDG-PET. This first PET exam allowed for randomizing the animals into three treatment groups according to infarct size. This randomization was crucial to avoid any bias due to different infarct sizes in experimental groups. Infarct size is indeed a major determinant of LV remodelling. A three-point classification was used to determine MI size, as previously described by our team [10]: small MI (no more than 3 among the 17 LV segments), moderate MI (4 to 5 segments) and large MI (at least 6 MI segments). Groups were constituted in order to exhibit equivalent rates of rats with small, moderate and large MI. In each of the three groups, rats were assigned to treatment with NaCl (control group; *n* = 7) or 2-chloroadenosine (2 mg/kg/day), a stable analogue of adenosine, which was given alone (CADO group; *n* = 8) or with 8-sulfophenyltheophilline (10 mg/kg/day), an antagonist of adenosine receptors (8-SPT group; *n* = 8). NaCl, CADO and 8-SPT (Sigma-Aldrich, Bornem, Belgium) were administered intraperitoneally twice daily for 2 months, starting 7 days after the date of LAD occlusion.

### ^18^ F-Fluorodeoxyglucose PET

Two days, 1 month and 2 months after surgery, LV function and infarct size were assessed *in vivo* by FDG-PET. As already described in detail elsewhere [10,13], rats received an oral pre-medication of 50 mg/kg of acipimox, a potent nicotinic acid derivative yielding high-quality cardiac FDG-PET images. Around 74 MBq of ^18^ F-FDG (IBA, Nancy, France) were injected intravenously under a transient anaesthesia with isoflurane. A 16-min recording was started 1 h later on a high-resolution dedicated small animal PET system (Inveon, Siemens, Knoxville, TN, USA) and under continuous anaesthesia by isoflurane. Images were reconstructed in 16 cardiac intervals with a 3-D ordered subset expectation maximization algorithm leading to a voxel size of 0.8 × 0.4 × 0.4 mm.

FDG uptake was determined on the set of collapsed short-axis slices in each segment from the 17-segment LV division from the American Heart Association [14] with the QGS software [15]. LV end-diastolic volume (EDV), LV end-systolic volume (ESV) and ejection fraction (EF) were obtained from the set of contiguous ECG-triggered short-axis slices with the QGS software [15,16]. The accuracy of these volume measurements was previously demonstrated on phantoms from LV rats above the level of 100 μL, corresponding to the lower limit for the LV end-systolic volume in adult rats [13]. The QGS software was also used for assessing the contractility of each of the 17 LV segments according to the percentage of systolic increase in myocardial counts [15]. This parameter is strongly linked to the percentage of myocardial thickening with both myocardial SPECT and TEP imaging techniques [17,18].

As previously described and validated [10], all segments for which the average FDG uptake was very low (<50% of the maximal voxel value) could be considered as predominantly necrotic, and the percentage of such segments was used to assess the extent of MI areas. For the segmental analysis of LV contractility, MI segments were defined as those showing a very low FDG uptake (<50% of the maximal voxel value) on the entire segment area, remote segments were defined as those which were not adjacent to any segment showing such a low uptake, and the remaining segments were considered to be within the border zone.

The average heart rate value during PET acquisition was extracted from the list mode recording data. Systolic blood pressure was calculated as the mean value of four recordings by the tail-cuff method during the PET acquisition (AD Instruments, Powerlab, Paris, France).

### Histological analyses

Rats were sacrificed 1 to 2 days after the last 2 months of PET acquisition with an overdose of sodium pentobarbital. The hearts were excised, snap frozen in liquid nitrogen, fixed and embedded in optimal cutting media (VWR, Fontenay-sous-Bois, France). Contiguous sections (8 μm), orientated along the vertical or horizontal long axis of the LV (depending on infarct location), were obtained in a cryostat at −22°C and stored at −80°C until analysis.

The degree of fibrosis in heart sections was assessed by Masson's trichrome staining. The collagen volume fraction was measured while omitting fibrosis of the perivascular, epicardial and endocardial areas [19]. The fibrosis area in the border zone was measured in three random fields per heart section (×400 magnification) by dividing the area of collagen to the total area and using ImageJ version 1.42 (http://rsbweb.nih.gov/ij/index.html).

Haematoxylin and eosin staining was performed to assess the cross-sectional area of cardiomyocytes in heart sections. For each section, at least five cardiomyocytes sectioned transversely were randomly chosen, and their area was quantified using ImageJ software. The average area was calculated for each experimental group.

Immunohistochemical staining was performed for annexin-5 apoptosis marker using a rabbit polyclonal antibody (Abcam, Cambridge, UK). Alexa Fluor® 635-coupled goat anti-rabbit antibody was used as secondary antibody (Invitrogen, Merelbeke, Belgium). Technical controls without primary antibody were performed for each marker to ensure staining specificity. DAPI was used to reveal nuclei. Images were recorded on a confocal microscope (Zeiss Laser Scanning Microscope LSM 510, Carl Zeiss Microscopy, Oberkochen, Germany) with a × 400 magnification using the LSM 510 META software (Carl Zeiss Microscopy, Oberkochen, Germany).

### Statistical analysis

Analyses were performed using the SAS R9.3 software (SAS Institute, Cary, NC, USA), and the two-tailed significance level was set to *p* < 0.05.

Pairwise comparisons of quantitative variables between the CADO or 8-SPT group and the control group were carried out using the Mann-Whitney test, and corresponding variables were expressed with mean ± standard error of the mean (SEM).

Segmental contractility was analysed over time using repeated measures mixed ANOVA: (1) with treatment group and segment location as fixed factors and (2) with rat as a random factor nested within the ‘treatment’ variable (all segments from a single rat received the same treatment). The results of these analyses were expressed as adjusted means ± SEM. Validity conditions of the models were thoroughly checked (normality of residuals, homoscedasticity and absence of collinearity or interaction). These global analyses were conducted for assessing, first, the effect of CADO alone (CADO vs. control) and, thereafter, the effect of CADO associated with 8-SPT (8-SPT vs. control). In order to preserve the overall 5% alpha error rate, the comparisons of CADO or 8-SPT vs. control were carried out at the 2.5% level.

## Results

### Early FDG-PET imaging and randomization

**Figure 1:**
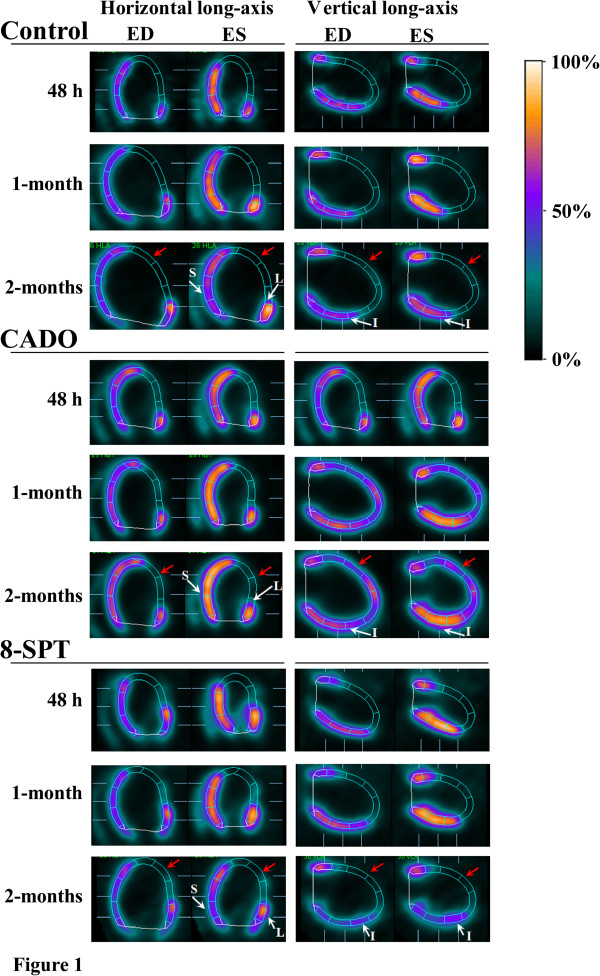
**Examples of FDG-PET images at 48 h, 1 month and 2 months in representative cases.** Horizontal and vertical long-axis slices are shown at both end-diastole (ED) and end-systole (ES), and segmental contractility is assessed through the increase in myocardial counts between ED and ES. Counts are represented through a colour scale, which is displayed on the right side of the figure and where the maximal count level (100%) is represented in white, 50% is in blue and less than 10% is in black. The change in the colour of myocardial walls from ED to ES images, which reflects contractile function, was marked outside the MI area (white arrows) for the three rats at baseline. This change was unaffected at 2 months for CADO rats, but was clearly affected for control and 8-SPT rats. Global LV remodelling was similar in the three experimental groups, with expansions of infarct areas (red arrows) and increases in LV volumes.

Representative FDG-PET images obtained at 48 h, 1 month and 2 months are shown in Figure 1. On the baseline FDG-PET scans recorded 2 days after coronary occlusion, necrotic segments were observed in all 23 animals. According to the three-point classification of MI size (small, moderate and large MI) [10], rats were subsequently randomized into three treatment groups: control (*n* = 7), CADO (*n* = 8) and 8-SPT (*n* = 8). Thus, the mean infarct size was equivalent between the three groups (Table 1). The mean baseline values of LV volumes, LV ejection fraction, heart rate, blood pressure and body weight were also similar between the three groups (Tables 1 and 2).

**Table 1:** Cardiac FDG-PET parameters

	**Control (*n*** **= 7)**	**CADO (*n*** **= 8)**	**8-SPT (*n*** **= 8)**
48 h			
Extent of MI area (% of LV)	35 ± 6	32 ± 15	32 ± 21
EDV (μL)	412 ± 61	397 ± 57	441 ± 66
ESV (μL)	239 ± 48	230 ± 43	257 ± 74
EF (%)	42 ± 7	42 ± 6	42 ± 7
1 month			
Extent of MI area (% of LV)	32 ± 9	29 ± 14	33 ± 23
Change from 48 h	−3 ± 7	−3 ± 4	1 ± 7
EDV (μL)	600 ± 95	576 ± 111	666 ± 175
Change from 48 h	188 ± 76	179 ± 120	225 ± 160
ESV (μL)	353 ± 67	324 ± 106	428 ± 185
Change from 48 h	114 ± 61	94 ± 112	171 ± 149
EF (%)	42 ± 2	45 ± 9	38 ± 12
Change from 48 h	0 ± 8	3 ± 10	−5 ± 7
2 months			
Extent of MI area (% of LV)	30 ± 11	26 ± 15	32 ± 27
Change from 48 h	−5 ± 9	−7 ± 8	0 ± 9
EDV (μL)	692 ± 132	637 ± 151	780 ± 232
Change from 48 h	280 ± 113	241 ± 158	339 ± 212
ESV (μL)	432 ± 120	379 ± 149	530 ± 210
Change from 48 h	19 ± 99	149 ± 145	273 ± 174
EF (%)	38 ± 5	42 ± 8	34 ± 10
Change from 48 h	−4 ± 6	0 ± 8	−9 ± 6

**Table 2:** Body weight, heart rate and blood pressure

	**Control (*n*** **= 7)**	**CADO (*n*** **= 8)**	**8-SPT (*n*** **= 8)**
48 h			
Body weight (g)	312 ± 25	307 ± 35	307 ± 20
Systolic blood pressure (mmHg)	104 ± 3	107 ± 8	102 ± 7
Heart rate (bpm)	419 ± 14	403 ± 38	422 ± 21
1 month			
Body weight (g)	353 ± 22	332 ± 30	332 ± 27
Change from 48 h	41 ± 15	25 ± 14	25 ± 22
Systolic blood pressure (mmHg)	106 ± 4	106 ± 4	104 ± 4
Change from 48 h	2 ± 4	−2 ± 8	2 ± 7
Heart rate (bpm)	374 ± 31	338 ± 35*	335 ± 48
Change from 48 h	−45 ± 36	−66 ± 69	−87 ± 60
2 months			
Body weight (g)	402 ± 12	363 ± 26*	376 ± 45
Change from 48 h	90 ± 22	56 ± 29	69 ± 43
Systolic blood pressure (mmHg)	104 ± 4	104 ± 3	104 ± 5
Change from 48 h	0 ± 5	−3 ± 6	2 ± 10
Heart rate (bpm)	383 ± 22	338 ± 33*	353 ± 39
Change from 48 h	−36 ± 27	−66 ± 44	−68 ± 36

*p < 0.05 for unpaired comparisons vs. control group.

### Evolution of body mass, hemodynamic parameters and global LV function

During the 2-month follow-up, all rats showed an increase in body weight, a decrease in heart rate and a stable level of systolic blood pressure. At 2 months, however, body weight and heart rate were slightly but significantly lower in CADO-treated rats than in control rats (Table 2).

As detailed in Table 1, control rats exhibited marked LV remodelling during the 2-month follow-up, as attested by increased EDV and ESV, and decreased EF. These changes were not significantly different in the CADO group or in the 8-SPT group, even though there were trends toward a beneficial effect on LV volumes and EF in the CADO group and a detrimental effect on LV volumes and EF in the 8-SPT group (Table 1).

### Evolution of segmental contractility

Seventeen segments were analysed in each of the 23 animals, leading to a total of 391 analysed segments. On the 2-day FDG-PET exams, 43 segments were totally necrotic (MI segments), 109 were considered as remote segments and the 239 remaining segments were considered to be within the border zone. Myocardial thickening, assessed through the percentage of systolic increase in myocardial counts, exhibited a continuous decline between remote (40% ± 9%), border zone (23% ± 13%) and MI (12% ± 4%) segments (*p* < 0.001).

Analyses of variance revealed that the evolution of myocardial thickening over 2 months was enhanced in CADO-treated rats, compared to control rats, with a significant interaction by segment location (remote, border zone or MI area) (*p* = 0.03). In contrast, no difference was observed when 8-SPT rats were compared to control rats using the same model.

**Figure 2:**
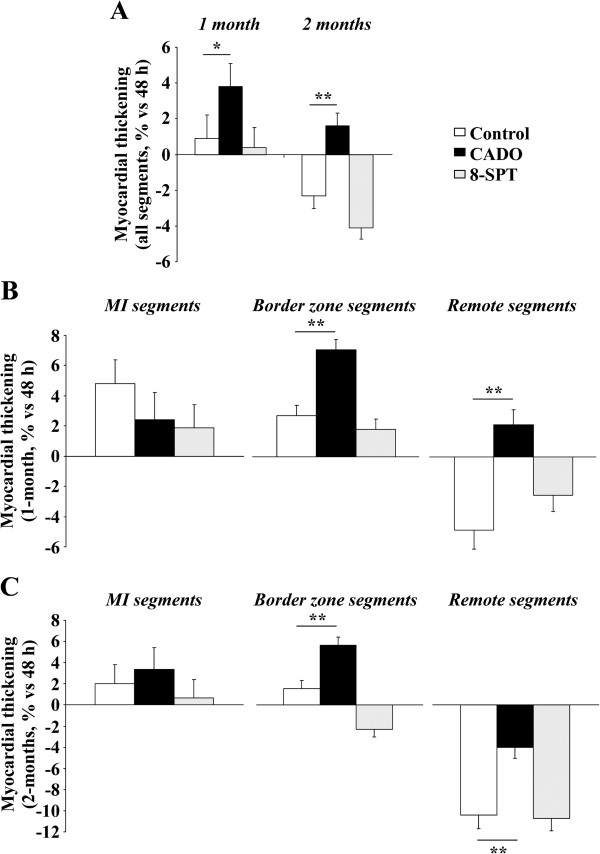
**CADO treatment improves segmental contractile function in remote regions after MI.** The evolution of segmental contractility in the three experimental groups is expressed as the change from 48 h to 1 and 2 months of the percentage of myocardial thickening. **(A)** Global evolution of segmental contractility (i.e. whole heart). **(B, C)** Evolution of segmental contractility after 1 and 2 months according to segment location (MI, border zone or remote area). Data are adjusted mean ± SEM. *p < 0.01; **p < 0.001.

Changes in segmental contractility from baseline were also compared between the three groups at the specific time-points of 1 and 2 months, as detailed in Figure 2. The main observation was that, on average, the contractile function of the overall segments from CADO rats was enhanced when compared to controls at 1 and 2 months (Figure 2A). After 1 month, the increase of myocardial thickening was +3.8% ± 0.7% in the CADO group compared to +0.9% ± 0.7% in the control group (*p* < 0.01). After 2 months, contractile function was impaired in control rats, as attested by a decrease of myocardial thickening of −2.3% ± 0.8%, and preserved in CADO-treated rats (+1.6% ± 0.8%, *p* < 0.001). This effect of CADO was blunted by 8-SPT (Figure 2A).

Interestingly, the beneficial effect of CADO on contractile function varied according to segment location, both after 1 month (Figure 2B) and after 2 months (Figure 2C). In particular, at 2 months, this effect was obvious (1) in the border zone where CADO rats exhibited a significant increase in contractile function (+5.6% ± 0.8%), whereas control rats did not (+1.5% ± 0.8%, *p* < 0.001 vs. CADO), and (2) in remote segments where a decline in contractile function was documented in all groups but to a lower extent in the CADO group (−4.0% ± 1.0%) than in the control group (−10.4% ± 1.3%, *p* < 0.001 vs. CADO). By contrast, the contractile function of MI segments was stable and comparable in the two groups at 2 months (CADO +3.4% ± 2.0% vs. control +2.0% ± 1.8%, *p* = NS).

The beneficial effect of CADO on the contractile function of the border and remote segments was lost for rats additionally treated with 8-SPT (Figure 2A,B).

### Fibrosis, cardiomyocyte hypertrophy and apoptosis

**Figure 3:**
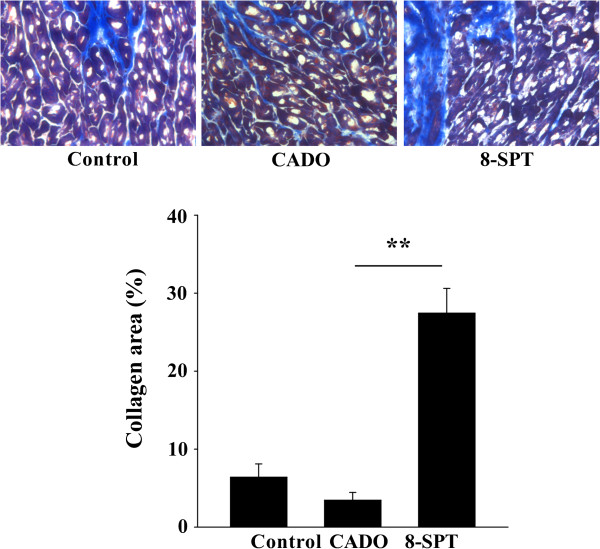
**8-SPT treatment aggravates cardiac fibrosis.** Upper panel: cardiac sections of non-infarcted areas of the left ventricle stained with Masson's trichrome. Representative pictures from the three experimental groups are shown. Magnification, ×400. Lower panel: quantitative analysis of collagen volume fraction in non-infarcted areas. Results are mean ± SEM. **p < 0.001.

We examined the extent of fibrosis outside the MI areas (Figure 3). CADO appeared to reduce fibrosis in remote areas, when compared to controls, although this difference did not reach the level of statistical significance. However, this fibrosis was markedly increased by the administration of 8-SPT (eightfold increase compared to the CADO group, *p* < 0.001).

**Figure 4:**
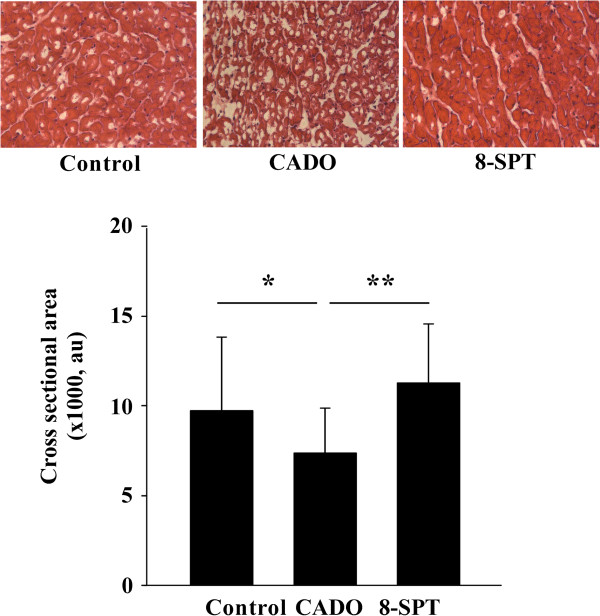
**CADO treatment limits cardiomyocyte hypertrophy.** Upper panel: representative histological sections stained with haematoxylin and eosin in remote areas showing cardiomyocyte hypertrophy. Lower panel: quantitative analysis of the cross-sectional area of cardiomyocytes from remote areas. Cardiomyocyte hypertrophy was decreased in CADO-treated rats compared to control rats. Results are mean ± SEM. au, arbitrary units. *p < 0.005; **p < 0.001. Magnification, ×400.

The cardiomyocyte cross-sectional area in healthy parts of the heart was lower in the CADO than in the control group, and this effect was blunted by 8-SPT (Figure 4).

**Figure 5:**
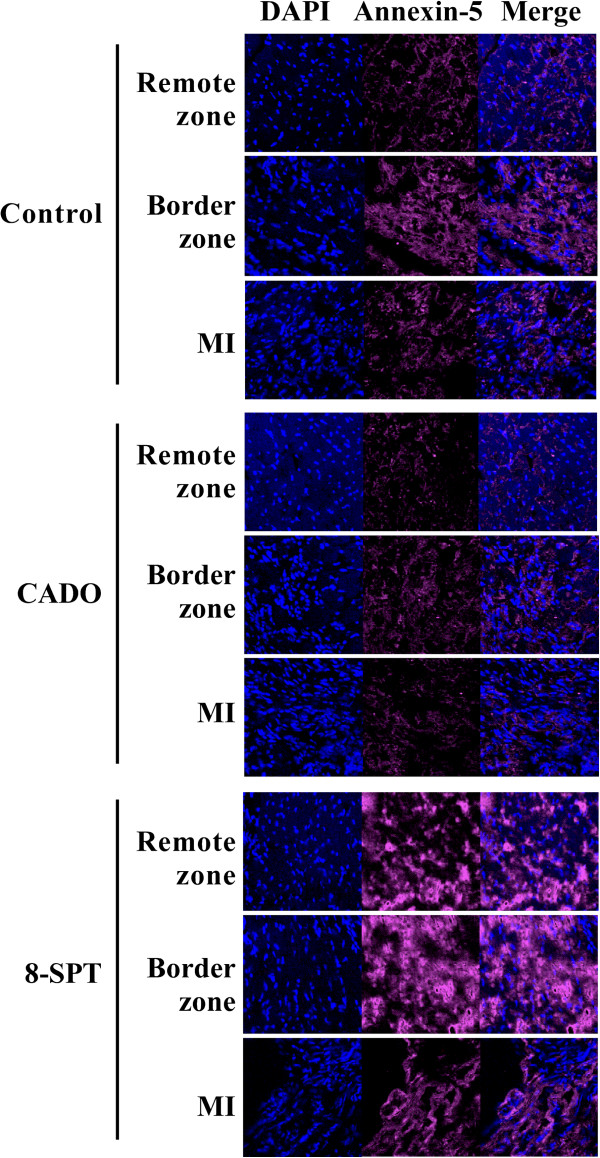
**CADO treatment reduces apoptosis.** Representative pictures of annexin-5 immunostaining in the remote, border and infarct (MI) zones are shown. Annexin-5 staining appears in pink, and nuclei are coloured in blue by DAPI. CADO reduced apoptosis and 8-SPT exacerbated apoptosis, particularly in the border zone. Magnification, ×400.

Annexin-5 staining was reduced in CADO rats compared to control rats and was robustly increased by 8-SPT (Figure 5). These differences were evident in the remote and border zones.

Therefore, activation of adenosine receptors reduces MI-induced fibrosis, cardiomyocyte hypertrophy and apoptosis.

## Discussion

Using FDG-PET in a rat infarct model, the present study shows that adenosine provides beneficial effects within the remote and border zones, but not within the MI area. However, these effects have a limited impact on global LV remodelling.

We have previously shown that the method of acipimox-enhanced FDG-PET provides cardiac images of a very high quality in rats [13], as well as accurate assessments of LV function and of MI size and location in the experimental model of LAD occlusion [10]. In the present study, the FDG-PET exams, performed 48 h after surgery, allowed for randomizing the animals into different treatment groups according to initial infarct size. This size, which is highly variable in the rat model, constitutes a main determinant of subsequent LV remodelling in the rat model [10]. Therefore, such a pre-therapeutic randomization was critical for providing an accurate comparison with the control group. This was not done in the previous echocardiographic study of long-term adenosine A2B receptor stimulation [8].

In addition, the FDG-PET technique allowed for a separate survey of the contractile function of the MI, remote and border segments. These data showed that treatment with CADO was beneficial on the contractile function of the segments lying outside the MI area. CADO treatment inhibited the progressive decline in the contractile function of remote areas and enhanced the contractile function within the border zone. These effects were truly attributable to the stimulation of adenosine receptors since they were abolished when a pan-antagonist of adenosine receptors was added to CADO treatment (8-SPT group).

The observation of a protective effect in the border zone is clinically relevant since the border zone is considered as a main therapeutic target after MI. For instance, injection of stem cells into the border zone has been shown to enhance vascular density and contractile performance in animals [20]. Similar results were achieved by the co-expression of vascular endothelial growth factor and angiopoietin-1 in the infarct border zone [21]. In addition, we have previously shown that the perfusion of the infarct border zone was enhanced after the injection of stem cells within the infarct core [22]. However, these therapeutic approaches are not easy to apply, whereas the present study shows that a simple pharmacological treatment is able to improve myocardial contractility.

The exact mechanism by which the stimulation of adenosine receptors improves contractile function in the border and remote zones remains to be fully defined. We observed a mild lowering of heart rate after 2 months of CADO treatment, but this is unlikely to fully explain the cardioprotective effects of CADO. Previous *in vitro* experiments from our group have shown that adenosine regulates multiple pathways involved in LV remodelling, such as inflammation, angiogenesis and extracellular matrix turnover [23-26]. In the present *in vivo* study, we further extend the cardioprotective properties of adenosine to apoptosis, consistent with a previous report by Simonis et al. [27], fibrosis [8] and cardiomyocyte hypertrophy [28]. All together, these results suggest that therapeutic targeting of adenosine receptors may have multiple cardioprotective effects.

Angiotensin-converting enzyme (ACE) inhibitors and beta-blockers are currently used to limit LV remodelling after MI. Although their effects on global function and on the limitation of infarct expansion are well documented [29], their effects in the border zone are still poorly known. Beta-blockers may only have a limited effect in the border zone because of multiple defects in the membrane beta-adrenergic receptor complex in the infarcted heart [30]. Our results, showing a preservation of contractile function in remote and border zones, but no effect on the infarct area, suggest that adenosine may target other signalling pathways than ACE inhibitors and beta-blockers. Whether adenosine exerts complementary cardioprotective effects to ACE inhibitors and beta-blockers could be the subject of further studies.

In our study, CADO was unable to significantly protect the heart from the development of adverse LV remodelling, as assessed by LV volumes and EF. These results differ from those of Wakeno and colleagues [8], where a selective adenosine A2B receptor agonist was reported to prevent LV remodelling after MI. In that study, LV function was assessed in only two dimensions by echocardiography and there was no documentation that the different experimental groups had equivalent infarct size before treatment. A2B receptors play critical roles in the reduction of remodelling and in the revascularization of the infarcted heart induced by mesenchymal stem cells [31]. Activation of A2B receptors inhibits apoptosis after MI [27]. However, a specific blockade of A2B receptors has been also shown to limit LV remodelling after MI [32]. The cardioprotective properties of A2B receptors are therefore complex and have been recently reviewed [33].

The roles of A1 and A3 receptors are also multiple and complex in this setting. Initial studies by Matherne and colleagues indicated that activation of the A1 adenosine receptor protects from myocardial ischemia [34]. This observation was subsequently confirmed by other groups (reviewed in [35]). The failure of CADO, a preferential A1 receptor agonist, to protect from LV remodelling in our study might be related to A3 activation, which has shown deleterious cardiac effects [36]. On the opposite, the A3 receptor has also cardioprotective properties [37].

Finally, all four sub-types of adenosine receptors appear to have the potential to exert cardioprotective effects after ischemia. Adenosine has multiple effects on several components of LV remodelling such as inflammation, fibrosis, angiogenesis, apoptosis, etc. These effects rely on the sub-type of adenosine receptors present on the cell surface. It remains to be determined whether the activation or blockade of several sub-types of adenosine receptors has superior anti-remodelling effects than the preferential activation of one receptor. It will also be interesting in future studies to address the cardioprotective effects of adenosine in a model of ischemia/reperfusion, which more closely resembles the clinical MI setting.

## Conclusions

We have shown that, when administrated long-term after MI, adenosine exerts beneficial effects on the myocardial contractility of remote and border zones, but with limited impact on global LV remodelling. Further studies are required to determine the therapeutic potential of this observation.

## Competing interests

The authors declare that they have no competing interests.

## Authors' contributions

MB performed the animal experiments and participated in the data analysis and manuscript drafting. FM, SP and HB participated in the PET exams and image analysis. JZ participated in the *ex vivo* experiments. RF performed the statistical analysis. GK and DW participated in the study design and manuscript drafting. YD and PYM supervised the study and drafted the manuscript. All authors read and approved the final manuscript.
